# Rotifers and lower crustaceans from South-western Iceland

**DOI:** 10.3897/BDJ.4.e7522

**Published:** 2016-03-02

**Authors:** Vesela V. Evtimova, Ivan S. Pandourski

**Affiliations:** ‡ Institute of Biodiversity and Ecosystem Research, Bulgarian Academy of Sciences, Sofia, Bulgaria

## Introduction

Iceland is one of the countries with the highest freshwater availability according to UNEP's Vital water graphics (http://www.eoearth.org/view/article/152861/). Additionally, being an island, it is rich in coastal brackish and saline aquatic habitats. However, little is known about the microcrustaceans and rotifers inhabiting these numerous habitats. The freshwater fauna of Iceland is relatively understudied compared to the fauna of adjacent marine ecosystems. Exhaustive sampling of deep-sea fauna was conducted within the inter-Nordic BIOICE project. As a result, Apostolov (2011) recorded 32 copepod harpacticoids of which 20 are new for the fauna of Iceland.

The first data on freshwater microinvertebrate fauna of Iceland date back to the 19^th^ century (Guerne and Richard 1892a, Guerne and Richard 1892b). The first study on the rotifer fauna from the middle of the 20^th^ century listed 59 species or subspecies (Bartoš 1951). The majority of the available studies on inland water bodies focused on large lakes: Mývatn in the north-east (Örnólfsd and Einarsson 2004, Adalsteinsson 1979, Jónasson 1979, Lindegaard 1979); and Thingvallavatn (Antonsson 1992) and Kerið Lakes (Evtimova et al. 2014) in the south-west of the country. Recently scientists have become increasingly interested in the inland freshwater copepods and cladocerans from small freshwater bodies (Novichkova et al. 2014, Scher et al. 2000). Data on observed morphological variability and teratology of lower crustacean in subpolar environments, including Iceland, were presented by Sinev et al. (2012), Pandourski and Evtimova (2009), Pandourski and Evtimova (2006), Pandourski and Evtimova (2005). These aberrations affected the fifth pair of legs in calanoids, the posterior part of the body in cyclopoids, or the head and antennule in cladocerans.

Our study presents data on taxa composition of Rotifera, Cladocera, and Copepoda in various aquatic habitats from South-western Iceland, including marine interstitial, wet bryophytes, springs, brackish and freshwater ponds and lakes.

## Materials and Methods

**Figure 1.**
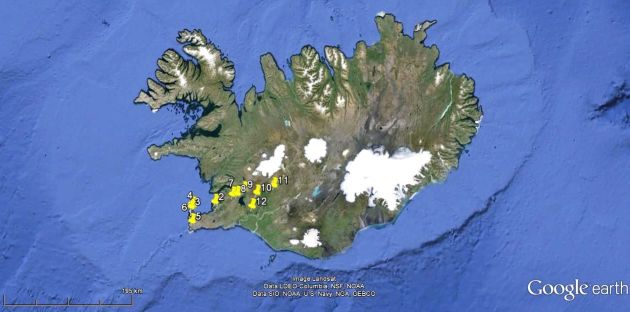
Map of Iceland with sampling locations from 1 to 12. For site numbers please see Table 1.

Samples were collected from various aquatic habitats from South-western Iceland. The sampling sites included marine interstitial habitat, puddles, swamps, freshwater or brackish lakes (Table 1, Fig. 1). Rotifers and lower crustaceans were collected using a qualitative plankton net (type “Apstein”, mesh size 38 µm) and a hand-held plankton net (mesh size 40 µm). The hand-held plankton net was used for sieving the sand and rinsing the bryophytes in order to collect the invertebrates inhabiting these substrata. The material was fixed in 70% ethanol.

The specimens were mounted temporarily in a mixture of glycerin and ethanol and were identified to the lowest practicable level following Wallace and Snell (2010), Sørensen (2009), Segers (1995), Einsle (1993), Monchenko (1974), Manuylova (1964). Harpacticoids were identified by Dr Apostolov and presented in earlier works (Apostolov 2014, Apostolov 2007).

## Results

A total of 39 taxa from Rotifera, Cladocera, and Copepoda were recorded from South-western Iceland during our study. The most diverse were the rotifers with 21 taxa belonging to nine families and two orders. We found 11 taxa of copepods which belonged to five families from three orders, and seven taxa of cladocerans from three families. Twelve associated invertebrate taxa were also found in our samples Table 2.

*Keratella quadrata* (Müller, 1786) was recorded at five of the sampled localities, while the copepod *Eucyclops serrulatus* (Fischer, 1851) and the cladoceran *Alona affinis* (Leydig, 1860) were found at four and three of the sites, respectively. Twenty-eight taxa were recorded only at one of the 12 sampling locations. We recorded the highest diversity of rotifers and the lowest diversity of crustaceans from bryophytes near Öxaráfoss waterfall in Þingvellir National Park.

## Discussion

We present data on rotifers and lower crustaceans from 12 aquatic habitats. For two of the stations (6 and 7), the lakes Sikið and Leirvogsvatn, we present the first data on zooplankton, and possibly also the first data for some of the smaller habitats (e.g. stations 3, 4, 5, 11). The majority of the recorded taxa either have a cosmopolitan distribution or are previously known from Iceland. For three of the recorded species we found no prevoius records in the available literature from Iceland: the rotifers Trichocerca cf. mucosa (Stokes, 1896) and *T. vernalis* (Hauer, 1936), and the copepod *Cyclopina gracilis* Claus, 1862. Rotifera dominated the sampled water bodies, followed by Copepoda and Cladocera. The most frequent taxon was the rotifer *Keratella quadrata*, previously recorded from Iceland by Bartoš (1951)​. All of the recorded rotifer species have a cosmopolitan distribution.

Many of the cladoceran taxa we recorded are frequently found in the arctic region. *Acroperus harpae* (Baird, 1835) is typical for the littoral fauna of freshwater lakes from the Holoarctic region (Novichkova et al. 2014, Sinev et al. 2012). Arctic populations of *Macrothrix hirsuticornis* Norman & Brady, 1867 are known to have high densities of specimens that are characterised with longer bodies and greater number of eggs per female (Meijering 2003, Margaritora and Usai 1983, Meijering 1979). *Macrothrix hirsuticornis* and *Alona quadrangularis* (Müller, 1785) are widely distributed and often are found in arctic regions and similar environments, likely owing to the resistance of their diapausing eggs to very low temperatures (Meijering 2003). We found these two species in permanent freshwater lakes (stations 6, 8, and 10).

All of the freshwater cyclopoid crustaceans recorded have cosmopolitan distribution and have been previously recorded from Iceland. We found only one marine copepod *Cyclopina gracilis* Claus, 1862. It is very common in the North Atlantic Ocean (Carey 1992, Grainger and Mohammed 1991, Mohammed and Neuhof 1985) but previously has not been reported from Iceland. The dominant cyclopoid in our samples was *Eucyclops serrulatus* (Fischer, 1851). *Cyclops abyssorum* Sars, 1863 is known to be among the dominant copepods in the large Icelandic lakes and is an important structural element for their zooplankton assemblages (Novichkova et al. 2014, Antonsson 1992). According to Larsen and Røen (1964) and Scher et al. (2000) another common cyclopoid for Iceland is *Megacyclops viridis* (Jurine, 1820). We found both *C. abyssorum* and *M. viridis* as well but only from shallow freshwater lakes (sites 6 and 8, correspondingly).

The two species of the harpactocoid genus *Bryocamptus* we recorded are associated with wet mosses (Evtimova et al. 2014, Apostolov 2007). *Nitokra spinipes* Boeck, 1865 can tolerate changes in salinity (Apostolov 2014) and was found from both brackish and freshwater habitats (sites 2 and 3).

## Conclusions

This manuscript presents faunistic data on microinvertebrate aquatic fauna, including new species records, from an understudied region where detailed data are still scarce. We found 39 taxa from 12 sites, and three of the recorded taxa are new for Iceland. Moreover, here we present first data on the zooplankton of Sikið and Leirvogsvatn Lakes. Future studies in the region would likely further enrich our knowledge on the composition and origin of microinvertebrate aquatic fauna of the island.

## Figures and Tables

**Table 1.** Locations and dates of sampling with coordinates and notes on water body type, habitat and substratum.

**Site**	**Date**	**Collection method**	**Habitat/ substratum sampled**	**Notes**	**Coordinates**
**No.**					
1	02.07.2004	Sieving	Marine interstitial, coarse sand	Garðskagaviti lighthouse; low tide	64°04'57.68"N, 22°41'36.08"W
2	08.07.2004	Hand-held net	Brackish lake, water column	Bessastaðatjörn Lake, coastal, shallow, coarse volcanic sand, macrophytes;	64°06'26.02"N, 21°59'43.79"W
3	29.06.2004	Hand-held net	Freshwater swamp, scraping overgrown stones	Small, c/a 200 m from Sandgerði Marine Centre	64°02'41.29"N, 22°42'45.64"W
4	29.06.2004	Hand-held net	Freshwater swamp, near the bottom	Small, beside Sandgerði Marine Centre, towards the sea; polluted	64°02'42.08"N, 22°42'45.14"W
5	30.06.2004	Hand-held net	Puddle overgrown by grass	Beside Sandvíkurtjörn Lake	63°51'14.90"N, 22°41'21.68"W
6	04.07.2004	Zooplankton net	Freshwater lake, water column	Sikið Lake; west of Garður Village	64°04'18.20"N, 22°38'45.38"W
7	05.07.2004	Zooplankton net	Freshwater lake, water column	Leirvogsvatn Lake, stoney bottom, high transperancy, oligotrophic, no macrophytes	64°12'07.42"N, 21°27'44.05"W
8	05.07.2004	Zooplankton net	Freshwater lake, water column	Small shallow, c/a 5-6 km eastwards from Stardalur and 35 km north-east of Reykjavik	64°12'37.89"N, 21°19'23.27"W
9	05.07.2004	Rinsing	Bryophytes	Wet mosses near Öxaráfoss waterfall, Þingvellir National Park	64°15'56.50"N, 21°07'02.94"W
10	05.07.2004	Hand-held net	Freshwater lake, water column	Laugarvatn Lake, shallow, hot springs on its shores; Arnes County, Laugardalur Valley	64°13'06.26"N, 20°43'40.61"W
11	05.07.2004	Rinsing	Spring, bryophytes	Small peat spring, low water temperature	64°18'24.79"N, 20°12'20.82"W
12	05.07.2004	Zooplankton net	Freshwater lake, water column	Kerið Lake, neovolcanic crater lake; Grimsnes area	64°02'26.36"N, 20°53'05.50"W

**Table 2.** List of taxa recorded from various habitats in South-western Iceland. For site numbers (No) please see Table 1.

**Group**	**Taxon**	**Site No.**
** Rotifera **		
**Class Eurotatoria**		
Order Ploima		
Family Brachionidae		
	*Keratella americana* Carlin, 1943	8; 12
	*Keratella cochlearis* (Gosse, 1851)	7
	*Keratella quadrata* (Müller, 1786)	3; 4; 5; 6; 7
	*Keratella* sp.	3
	*Notholca acuminata* Ehrenberg, 1832	2
Family Lecanidae		
	*Lecane crenata* (Harring, 1913)	10; 12
	*Lecane* sp.	10
	*Lecane nana* (Murray, 1913)	9
	*Lecane* sp.	9
Family Asplanchnidae		
	*Asplanchna* sp.	7
Family Lepadellidae		
	*Colurella sulcata* (Stenroos, 1898)	12
	*Colurella* sp.	12
	*Lepadella* (s. str) sp.	10
	*Lepadella* sp.	4
Family Nothommatidae		
	*Cephalodella* sp.	9; 12
Family Euchlanidae		
	*Euchlanis dilatata* Ehrenberg, 1832	10
Family Proalidae		
	*Proales* sp.	11
Family Trichocercidae		
	Trichocerca cf. mucosa (Stokes, 1896)	6
	*Trichocerca vernalis* (Hauer, 1936)	8
	*Trichocerca* sp.	9
Order Flosculariaceae		
Family Trochosphaeridae		
	*Filinia terminalis* (Plate, 1886)	3
**Class Branchiopoda**		
Order Anomopoda		
Family Daphnidae		
	*Daphnia pulex* Leydig, 1860	6
Family Chydoridae		
	*Acroperus harpae* (Baird, 1835)	8
	*Alona affinis* (Leydig, 1860)	8; 10; 12
	*Alona quadrangularis* (Müller, 1785)	8; 10
	*Chydorus sphaericus* (Müller 1776)	6
	*Chydorus* sp.	7
Family Macrothricidae		
	*Macrothrix hirsuticornis* Norman & Brady, 1867	6
**Class Maxillopoda**		
Order Calanoida		
Family Temoridae		
	*Eurytemora velox* (Lilljeborg, 1853)	2
Order Cyclopoida		
Family Cyclopidae		
	*Acanthocyclops vernalis* (s. lat. Fischer, 1853)	9; 12
	*Cyclops abyssorum* Sars, 1863	6
	*Diacyclops bisetosus* (Rehberg, 1880)	3; 9
	*Eucyclops serrulatus* (Fischer, 1851)	6; 8; 9; 10
	*Megacyclops viridis* (Jurine, 1820)	8
	*Paracyclops fimbriatus fimbriatus* (Fischer, 1853)	8
Family Cyclopinidae		
	*Cyclopina gracilis* Claus, 1862	1
	Copepodites	1; 2; 6; 8; 10; 12
	Nauplii	2; 6; 7; 10; 12
Order Harpacticoida		
Family Canthocamptidae		
	Bryocamptus (Arcticocamptus) cuspidatus cuspidatus (Schmeil, 1893)	9; 11
	Bryocamptus (Bryocamptus) minutus (Claus, 1863)	12
Family Ameiridae		
	*Nitokra spinipes* Boeck, 1865	2; 3
**Class Ostracoda**		
	Ostracoda indet.	1; 6; 9; 11
**Associated fauna**		
	Amphipoda	2
	Acari	5; 1; 9
	Acari (Halacaridae)	5
	Olygochaeta	10
	Polychaeta	1
	Colembolla	5; 9
	Tardigrada	1; 9
	Gastropoda	1; 9
	Diptera larvae	2; 9; 11
	Diptera (Chironomidae) larvae	10; 12
	Nematoda	9; 10; 12

## Project

Taxonomy, faunistics and zoogeography of brackish and freshwater copepods (Crustacea) from the Reykjanes peninsula, Iceland (June 2004 – September 2007).

## Hosting institution

Sanðgerdi Marine Centre and the Institute of Biology, University of Iceland.

## Project

Taxonomy, faunistics and zoogeography of brackish and freshwater copepods (Crustacea) from the Reykjanes peninsula, Iceland (June 2004 – September 2007).

## Hosting institution

Sanðgerdi Marine Centre and the Institute of Biology, University of Iceland.
